# The association between diagnosis disclosure and adherence to antiretroviral therapy among adolescents living with HIV in sub-Saharan Africa: a protocol for systematic review and meta-analysis

**DOI:** 10.1186/s13643-020-01420-8

**Published:** 2020-07-14

**Authors:** Melkamu Merid Mengesha, Dessalegn Ajema, Awugchew Teshome, Abera Kenay Tura

**Affiliations:** 1 School of Public Health, College of Health and Medical Sciences, Haramaya University, P.O. Box 235, Harar, Ethiopia; 2 School of Public Health, College of Medicine and Health Sciences, Arba Minch University, Arba Minch, Ethiopia; 3 Department of Environmental Health Sciences, College of Health and Medical Sciences, Haramaya University, Harar, Ethiopia; 4 School of Nursing and Midwifery, College of Health and Medical Sciences, Haramaya University, Harar, Ethiopia

**Keywords:** Disclosure, Adherence, Antiretroviral therapy, Adolescents, Sub-Saharan Africa, Systematic review

## Abstract

**Introduction:**

More than eight in ten of the world’s 1.65 million adolescents living with human immunodeficiency virus (ALHIV) live in sub-Saharan Africa (SSA). Suboptimal adherence to antiretroviral therapy (ART) and poor viral suppression are reported among ALHIV which may in turn compromise the gains achieved so far. The evidence on whether knowing one’s own human immunodeficiency virus (HIV) status and self-disclosure to others benefit adherence to ART or not is inconclusive. This review aims to estimate the association between knowing one’s HIV status and self-disclosure on adherence to ART among ALHIV in SSA.

**Methods:**

Comprehensive search strings will be used to identify relevant observational studies published in English up to May 2020 in major databases: Excerpta Medica database (EMBASE), PubMed, and Ovid/MEDLINE. To access African studies and also to freely access subscription-based articles, the African Index Medicus (AIM) and the WHO HINARI databases will be searched. The AfroLib database will be searched to access the gray literature of African studies. We will use the COVIDENCE software for title/abstract screening, full-text screening, quality assessment, and data extraction. Two authors will independently screen retrieved articles, and a third author authorized to resolve conflicts will handle disagreements. The Joanna Briggs Institute’s (JBI) critical appraisal tools will be used to assess study quality. Appropriate statistical tests will be conducted to quantify the between studies heterogeneity and for the assessment of publication bias. We will check individual study influence analysis and also do subgroup analysis. The STATA version 14.2 will be used for statistical analysis.

**Discussion:**

A high-level adherence to ART is required to achieve adequate viral suppression and improve quality of life. Consequently, the evidence on how adherence to ART differs with knowledge of one’s own HIV status and self-disclosure may help guide interventions aimed at improving adherence to ART.

## Introduction

Globally, 1.65 million adolescents (10–19 years of age) were living with human immunodeficiency virus (HIV) in 2018, of whom 88% (1.46 million) were in sub-Saharan Africa (SSA) [1]. Improved coverage and access to antiretroviral therapy (ART) have changed the epidemiology of the HIV epidemic as children and adolescents transition to adulthood [2–4]. Despite significant gains in reducing HIV/AIDS-related mortality and new infections in other age groups [5], new HIV infections among adolescents show a rising trend in SSA with a particularly higher rate in the Eastern and Southern Africa [6].

Sustained improvement in survival, viral load suppression, and reduction in onward transmission depends on high levels of compliance to the daily ART dosing (> 95%) [7, 8]. Poor adherence to ART increases the risk of viral drug-resistance, reduces treatment efficacy, increases opportunistic infections leading to disease progression, and reduces future therapeutic options [9]. Research evidence indicates that as adolescents get older and parental supervision becomes low, adherence to their ART medication is becoming a problem [10]. A systematic review of literature on adherence to ART estimated that only 62.3% of ALHIV were adherent [9]. Williams et al. reported that the level of adherence among older adolescents was lower compared to younger children, 76% versus 83–89% [11]. Viral load was also ten times higher among non-adherent compared to adherent children and adolescents [11].

In a qualitative synthesis of factors impacting adherence to ART among ALHIV, Ammon et al. reported four themes of factors that affect adherence including patient, medication, caregiver, and health system-related factors [12]. Among the several factors (44 barriers and 29 facilitators) reported, most were patient-related—not being aware of the reasons for taking medications and lack of HIV status disclosure to other family members, peers, or partners [12]. However, a review by Nichols et al. found inconclusive evidence on the benefit of HIV status disclosure on adherence to ART [13].

A recent systematic review by Doat et al. on disclosure of HIV status reported that disclosure has both merits and demerits [14]. The disadvantages are often reported concerning children’s mental health and relationship status. Among the benefits reported include that disclosure created opportunities for adolescents to seek adherence and psychosocial support, take control of their health, and become free to communicate and ask questions when they need help [14]. Despite the existence of evidence that disclosure of children’s HIV status has benefit in HIV/AIDS care, the level of full disclosure remained very low in SSA, still being below 50% [15, 16].

Previous evidence from systematic reviews on the effect of disclosure on adherence to ART among ALHIV came from either qualitative studies [12] or cross-sectional studies that included infants and under-five children (whose adherence completely depends on their caregivers) [13], making it difficult to generalize results and hence design appropriate interventions for adolescents to improve adherence to ART. Therefore, it is important to study the level of adherence and the associated factors among this particular population, who are supposed to be at the center of the HIV-epidemic [17]. This systematic review and meta-analysis aim to pool estimates on the level of adherence and factors associated with adherence to ART among ALHIV in SSA. Besides, we plan to conduct a subgroup analysis by the type adherence measure used, disclosure form, and the route of HIV infection. The research questions this systematic review seeks to answer are as follows: What is the level of adherence to ART among ALHIV? Does disclosure of HIV status benefit adherence to ART among ALHIV in SSA?

## Methods

### Protocol and registration

This systematic review and meta-analysis will be conducted following the recommendation of the Preferred Reporting Items for Systematic Reviews and Meta-Analysis Protocols (PRISMA-P) guidelines [18]. This review protocol is registered in the International Prospective Register of Systematic Reviews (PROSPERO) system and can be accessed at https://www.crd.york.ac.uk/prospero/display_record.php?ID=CRD42020178084. See (Additional file 1) for the completed PRISMA-P checklist.

### Eligibility criteria

#### Study design/characteristics

Observational studies (analytic cross-sectional, case-control, and cohort) that reported the association between disclosure and adherence to ART among ALHIV will be considered for inclusion. In this review, we will consider studies conducted in sub-Saharan African countries and published in English up to May 2020.

#### Population

Studies involving adolescents (ages between 10 and 19 years) who acquired HIV either through perinatal infection or behaviorally and primary caregivers will be eligible. Primary caregivers are adults over the age of 18 years who live in the same household as the adolescent and able to report about the HIV/AIDS-related care that the adolescent receives.

#### Exposure

The primary exposure of interest is the disclosure of HIV status. Studies reporting adolescents own HIV status disclosure by caregivers and the adolescent’s onward disclosure of his/her HIV status to significant others including friends and families will be eligible. Informing adolescents of their own HIV status is a pre-requisite to onward or self-disclosure. However, full disclosure which is limited in the HIV/AIDS care in SSA [14] is defined when the adolescent knows the name of his/her illness (HIV and/or AIDS), received disease-specific information (for example, how the virus is transmitted and ways of prevention), and knows how they acquired the infection [19].

#### Comparators (controls)

In order to be eligible for inclusion, studies must compare the outcome in the exposed group (adolescents who received full disclosure of their HIV status) against the outcome in the unexposed group (adolescents who did not receive full disclosure of their HIV status).

#### Outcome

Studies reporting the prevalence of self-reported (both caregiver and adolescent based report) or objective measures of adherence to ART and effect sizes of the association between disclosure and adherence will be eligible for inclusion. Different methods of assessment of adherence to ART have been reported including medication event monitoring system (MEMS), pill counts, review of pharmacy records, or patient self-report [20]. Despite its limitation of overestimating adherence, self-reported measures of adherence still have clinical value in predicting viral load and hence screening for poor adherence [20, 21]. Consequently, in this review, we will consider primary studies reporting self-reported measures of adherence, MEMS, pharmacy-based measures, or mixed methods of adherence measures. Percent of medication doses taken (≥ 95%) will be used to designate optimal adherence.

### Data source and search strategy

A comprehensive search will be conducted in the major databases—PubMed/MEDLINE, Excerpta Medica database (EMBASE), and Ovid/MEDLINE—using key terms and Medical Subject Headings (MeSH) specifically designed for the respective databases. To access subscription-based articles and African-based studies, the World Health Organization HINARI database and the African Index Medicus (AIM) will be used, respectively. Furthermore, to access the gray literature of African-based studies, we will use the AfroLib database. The key terms that will be used to build the search strings include “adolescents,” “disclosure,” “self-disclosure,” “adherence,” “antiretroviral therapy,” and “sub-Saharan Africa.” Besides, a bibliographic search of identified articles and gray literature search will be done for identifying additional articles. In case of doubts and when additional information is sought, authors of primary studies will be contacted. The complete list of the search strategy and key terms is summarized in an additional file (Additional file 2).

### Data management and study selection

Articles retrieved from databases will be exported to EndNote version 9.1 citation manager and will then be exported to COVIDENCE, a software for a systematic review production tool for title/abstract screening, full-text screening, data abstraction, and quality assessment [22]. COVIDENCE removes duplicates and requires at least two reviewers for a vote to be made whether a given article should proceed to the next process or not. Accordingly, in the COVIDENCE system, screening of title and abstract of articles will be conducted independently for relevance by any two reviewers [MMM and AT/or AKT or DA]. Any of the two reviewers [MMM and AT/or AKT or DA] will also review the full-text screening of retained articles. A disagreement between the two reviewers will be resolved by a third reviewer authorized to resolve conflicts [MMT or AKT], and the decision will be final either to include or exclude an article.

### Data extraction and quality assessment

We will also conduct the data extraction and quality assessment by using COVIDENCE [22]. As the data extraction form and risk of bias assessment in COVIDENCE are not limited to the standard randomized controlled trials, options in the system can be customized to add categories that fit for observational studies. The PECOS format will be adopted to extract data. Two authors [MMM and AT] will independently extract data on study identification, methods, population, exposure, and outcome. As COVIDENCE provides options to choose among templates for quality assessment, we will use the custom template for the risk of bias assessment [22] and add assessment domains. Domains of the quality of studies retained for full-text review will be checked by the same authors [MMM and AT] independently using the Joanna Brigs Institute (JBI) critical appraisal tools for analytic cross-sectional study, prevalence study, case-control study, and cohort study as appropriate [23]. The JBI critical appraisal tool has eleven items to assess cohort studies, ten items to assess case-control studies, and eight items to assess analytic cross-sectional studies [23]. See (Additional file 3) for details on the Joanna Briggs Institute’s critical appraisal checklists for observational studies. Results of study quality assessment and data extracted will be exported in a preferred format for data synthesis and statistical analysis.

### Data synthesis and statistical analysis

The STATA version 14.2 will be used to pool estimates of the prevalence of adherence level and effect sizes on the association between disclosure and adherence to ART. With the assumption that the effect sizes estimated in different studies are not identical, but follow some distribution, we plan to implement the random-effects model using the method of DerSimonian and Laird, with the estimate of heterogeneity being taken from the inverse-variance fixed-effect model [24, 25]. For the meta-analysis of prevalence, a procedure in STATA, called *metaprop*, that performs the Freeman-Tukey double arcsine transformation, will be used to compute the weighted pooled estimate and perform back-transformation on the pooled estimate [26]. The random-effects model will also be used to combine the prevalence estimates. The model selection, however, will further be informed by the heterogeneity assessment. Study results will be descriptively summarized if there is a substantial heterogeneity to pool estimates.

The Higgins *I*^2^ statistic will be used to describe the percentage of the total variability in study estimates that is due to heterogeneity [27]. The *I*^2^ statistic values of 25%, 50%, and 75% would mean low, medium, and high heterogeneity, respectively [27]. Sources of the between studies heterogeneity will be assessed by a subgroup analysis. The potential variables we will consider for a subgroup analysis include the type of adherence measures used, the form of disclosure (caregivers tell their children the truth that they have HIV and the adolescent’s self-disclosure of HIV status to family members, peers, and to significant others), and the route of HIV infection (whether acquired HIV infection perinatally or behaviorally). To further assess whether these factors explain any observed heterogeneity of effect size estimates, we will conduct a meta-regression. We will also conduct a single study influence analysis to observe the effect of omitting a single study on the overall pooled effect estimate [28].

The publication bias, which represents the tendency to report positive findings [29], will be visually checked by inspecting the funnel plot and also objectively by using the Harbord’s regression test to statistically assess the asymmetry of the funnel plot [30]. The funnel plot is a graphical presentation of individual study effect estimates on the *x*-axis plotted against their sample sizes or precisions on the *y*-axis. In the absence of publication bias, the effect size estimates would distribute evenly around the pooled effect size with a greater variability for small studies [29]. We will use the contour-enhanced funnel plot to take advantage of distinguishing between publication bias and other causes of asymmetry which the standard method fails to do [31].

Summary of pooled estimates will be presented graphically in terms of forest plots and the visual assessment of publication bias. Study characteristics of original studies including study objectives, population studied, adherence measures used, adjustments for confounding, risk of bias, and major findings will be summarized and presented in a table.

## Discussion

This protocol describes a planned systematic review and meta-analysis to estimate the effect of HIV-status disclosure among ALHIV in SSA. Improved coverage and access to highly effective ART proved substantial contribution in changing a rapidly fatal disease into a chronic condition [2, 3]. Consequently, children and adolescents can survive longer and transition to adulthood [32]. To ensure the wellbeing of ALHIV and also reduce onward transmission through a sustained viral suppression, compliance to a lifelong ART is vital [33].

Although previous studies have shown suboptimal adherence to ART among ALHIV and identified several factors associated with adherence [9, 34], the effect of disclosure on ART adherence was inconclusive [13]. For instance, Grimsrud et al. reported that disclosure was associated with an increase in medication adherence, mediated through increased social support [33]. A review by Nichols et al. on the impact of disclosure on adherence to ART, however, found no conclusive evidence [13]. Once adolescents are aware of their HIV status, how their knowledge that they have HIV and self-disclosure to significant others including family members, friends or sexual partners, and teachers influence adherence to ART is not pooled quantitatively. This review aims to pool estimates on the adherence level of ALHIV and the effect of disclosure on adherence to ART.

Given the poor adherence to ART among ALHIV [9, 34], the pooled estimate of the association between disclosure and adherence to ART in SSA is needed to inform interventions towards achieving optimal adherence and thereby adequate viral suppression and improved quality of life.

## Supplementary information

**Additional file 1.** PRISMA-P 2015 Checklist.

**Additional file 2.**Search strings.

**Additional file 3.** JBI Critical Appraisal Checklist.

## Data Availability

Not applicable.

## Abbreviations

ALHIV: Adolescent living with human immunodeficiency virus
AIDS: Acquired immune deficiency syndrome
ART: Antiretroviral therapy
CINAHL: Cumulative Index of Nursing and Allied Health Literature
EMBASE: Excerpta Medica database
HIV: Human immunodeficiency virus
JBI: Joanna Briggs Institute
MeSH: Medical Subject Heading
SSA: Sub-Saharan Africa
UNAIDS: The Joint United Nations Program on HIV/AIDS
