# Supplementary material for: The association between diagnosis disclosure and adherence to antiretroviral therapy among adolescents living with HIV in sub-Saharan Africa: a protocol for systematic review and meta-analysis
Source: Syst Rev. 2020 Jul 14;9:160. doi: 10.1186/s13643-020-01420-8 (PMC7362460; doi:10.1186/s13643-020-01420-8)
Supplement: Supplementary file 2 — Additional file 2.Search strings. [file 13643_2020_1420_MOESM2_ESM.docx]

Search strings built for literature search in the respective databases for a systematic review and meta-analysis on the “The association between diagnosis disclosure and adherence to antiretroviral therapy among adolescents living with HIV in sub-Saharan Africa: A protocol for systematic review and meta-analysis.”

Key concepts: disclosure, self-disclosure, adherence, antiretroviral therapy, adolescents, sub-Saharan Africa

PubMed: (((africa south of the sahara[MeSH Terms]) AND ((adolescent[MeSH Terms]) OR adult children[MeSH Terms])) AND ((((((((medication adherence[MeSH Terms]) OR patient compliance[MeSH Terms]) OR anti-retroviral agents[MeSH Terms]) OR antiretroviral therapy, highly active[MeSH Terms]) OR adherence to antiretroviral therapy[Title/Abstract]) OR adherence to highly active antiretroviral therapy[Title/Abstract]) OR adherence to haart[Title/Abstract]))) AND ((((((((truth disclosure[MeSH Terms]) OR self disclosure[MeSH Terms]) OR disclosure) OR status disclosure[Title/Abstract]) OR HIV serostatus disclosure[Title/Abstract]) OR HIV status disclosure[Title/Abstract]) OR HIV diagnos*s disclosure))

Ovid (MEDLINE): (disclosure.mp. OR self disclosure.mp.) AND (adherence.mp. OR compliance.mp. OR Anti-Retroviral Agents/ or Antiretroviral Therapy, Highly Active/ or antiretroviral therapy.mp. or Anti-HIV Agents/) OR ART.mp. OR HAART.mp.) AND (adolescent*1.mp. OR teenage*.mp.) AND (Africa, Western/ or South Africa/ or Africa, Eastern/ or africa.mp. or "Africa South of the Sahara"/ or Africa, Central/ or Africa/ or Africa, Southern/)

EMBASE: ('interpersonal communication'/exp OR 'self disclosure'/exp OR 'disclosure'/exp OR 'status disclosure' OR 'hiv serostatus disclosure' OR 'hiv diagnos$s disclosure') AND ('medication compliance'/exp OR 'adherence to antiretroviral therapy' OR 'adherence to highly active antiretroviral therapy' OR 'adherence to haart' OR 'compliance to art' OR 'adherence to art' OR 'compliance to art drugs' OR 'hiv drug adherence' OR 'antiretroviral therapy') AND ('adolescent'/exp OR 'adult child' OR 'teenager') AND ('africa south of the sahara'/exp)

HINARI:((SubjectTerms:(disclosure)) OR (HIV status disclosure) OR (HIV diagnosis disclosure)) AND ((SubjectTerms:(adherence)) OR (adherence to ART) OR (antiretroviral therapy)) AND ((SubjectTerms:(adolesent)) OR (SubjectTerms:(adult children)) OR (SubjectTerms:(teenager))) AND ((SubjectTerms:(africa south of the sahara)) OR (SubjectTerms:(sub saharan africa)) OR (SubjectTerms:(africa, western)) OR (SubjectTerms:(africa, central)) OR (SubjectTerms:(africa, eastern)) OR (SubjectTerms:(africa, southern)))
